# Dog size and patterns of disease history across the canine age spectrum: Results from the Dog Aging Project

**DOI:** 10.1371/journal.pone.0295840

**Published:** 2024-01-17

**Authors:** Yunbi Nam, Michelle White, Elinor K. Karlsson, Kate E. Creevy, Daniel E. L. Promislow, Robyn L. McClelland, The Dog Aging Project Consortium

**Affiliations:** 1 Department of Biostatistics, University of Washington, Seattle, WA, United States of America; 2 Program in Molecular Medicine, University of Massachusetts Medical School, Worcester, MA, United States of America; 3 The Broad Institute of Harvard and MIT, Cambridge, MA, United States of America; 4 Bioinformatics and Integrative Biology, University of Massachusetts Chan Medical School, Worcester, MA, United States of America; 5 Department of Small Animal Clinical Sciences, Texas A&M University College of Veterinary Medicine & Biomedical Sciences, College Station, TX, United States of America; 6 Department of Laboratory Medicine and Pathology, University of Washington School of Medicine, Seattle, WA, United States of America; 7 Department of Biology, University of Washington, Seattle, WA, United States of America; Leibniz Institute on aging - Fritz Lipmann Institute (FLI), GERMANY

## Abstract

Age in dogs is associated with the risk of many diseases, and canine size is a major factor in that risk. However, the size patterns are complex. While small size dogs tend to live longer, some diseases are more prevalent among small dogs. In this study we seek to quantify how the pattern of disease history varies across the spectrum of dog size, dog age, and their interaction. Utilizing owner-reported data on disease history from a substantial number of companion dogs enrolled in the Dog Aging Project, we investigate how body size, as measured by weight, associates with the lifetime prevalence of a reported condition and its pattern across age for various disease categories. We found significant positive associations between dog size and the lifetime prevalence of skin, bone/orthopedic, gastrointestinal, ear/nose/throat, cancer/tumor, brain/neurologic, endocrine, and infectious diseases. Similarly, dog size was negatively associated with lifetime prevalence of ocular, cardiac, liver/pancreas, and respiratory disease categories. Kidney/urinary disease prevalence did not vary by size. We also found that the association between age and lifetime disease prevalence varied by dog size for many conditions including ocular, cardiac, orthopedic, ear/nose/throat, and cancer. Controlling for sex, purebred vs. mixed-breed status, and geographic region made little difference in all disease categories we studied. Our results align with the reduced lifespan in larger dogs for most of the disease categories and suggest potential avenues for further examination.

## Introduction

Age is the single greatest predictor of disease risk for most causes of mortality in both human and dog populations [1–3]. However, for many dog diseases, body size is a comparably important predictor of risk [1, 4, 5]. Companion dogs show considerable variation in longevity across size classes [6–8]. Between species of mammals, larger ones tend to live longer than smaller ones, while within species, smaller individuals tend to live longer than larger individuals [9, 10]. Accordingly, dogs from larger size classes tend to have a shorter lifespan. Additionally, different size classes of dogs tend to manifest with different diseases, and ultimately to die from different causes. For instance, larger breed dogs more often die as a consequence of musculoskeletal and gastrointestinal causes whereas smaller dogs die more frequently of endocrine causes [1, 2].

Jin *et al*. [11] studied a multimorbidity index (i.e., a count of the number of conditions a dog has experienced) and found that although the index increased steadily with age, the size of the dog had no significant effect on the index. This suggests that over time, dogs in larger size classes are not accumulating *more* conditions, but rather *different* conditions. An understanding of which conditions manifest differently across age and size could inform our understanding of size-related longevity differences.

The Dog Aging Project (DAP) provides a unique opportunity to investigate how the lifetime prevalence of different conditions varies across age and size in a large community-based population of companion dogs. Additionally, we explore whether the adjustment for the dog’s sex, breed status (purebred vs. mixed-breed status), and the census division defined by the dog’s residence address impacts this relationship [12–17]. Specifically, we use data from the “DAP Pack”, a collection of over 25,000 survey respondents from across the US.

## Materials and methods

### Data collection

The DAP was launched in [18]. Dog owners self-selected and could enroll one dog per household. Enrollment consisted of completing a web-based Health and Life Experience Survey (HLES) that elicited information from the dog owner on a wide array of topics. Because the Dog Aging Project is an Open Data project, survey instruments are made publicly available on GitHub: [https://github.com/dogagingproject/survey_instruments/tree/main/HLES/annotated_with_terra_names] and raw data are made publicly available annually through the Terra platform [https://dogagingproject.org/open_data_access/]. The University of Washington Institutional Review Board (IRB) deemed that recruitment of dog owners for the Dog Aging Project, and the administration and content of the DAP questionnaires, are human subjects research that qualifies for Category 2 exempt status (IRB ID no. 5988, effective 10/30/2018). No interactions between researchers and privately owned dogs occurred during recruitment of dogs to the project or completion of surveys; therefore, IACUC oversight was not required.

### Data description

The curated 2020 release of the HLES data contained 27,541 survey records collected on or before December 31st, 2020. The survey consisted of ten sections including dog demographics, health status, and owner demographics. In the health status section, dog owners were asked if their dogs have ever been diagnosed with various medical conditions regardless of the current status, within a set of pathophysiologic process or organ system categories. Pathophysiologic processes that often affect more than one organ system (e.g., cancer, trauma, infection, etc.) were presented at the beginning, to encourage owners to record such conditions in those categories. Organ system categories (e.g., kidney disease, ocular disease, etc.) were presented later in the survey. At the beginning of each category, a list of example diagnoses within that category was presented to prompt owner recall. For each disease category for which owners indicated the presence of a diagnosis, they then indicated specific condition(s) within that category, including an option of “other, please describe.” We analyzed thirteen disease categories of interest within which conditions were reported in 500 or more dogs. Categories that were considered include skin disorders, infectious or parasitic disease, orthopedic, gastrointestinal, ocular, ear/nose/throat (ENT), kidney/urinary, cancer, cardiac, neurologic, liver/pancreas, respiratory, and endocrine disorders. We did not analyze specific diagnoses chosen within each category.

Owners were asked to provide the year and month the dog was born if known or to provide an estimated age at the time they filled out the survey. Based on this calculated age, we categorized the dogs into puppies (<1 year), adolescents (1 to <3 years), young adults (3 to <7 years), older adults (7 to <11 years), or seniors (11+ years) at the time of survey completion. It is worth noting that while the labels of age range may not perfectly align with all breeds, we aimed to correspond them with the general lifespan status concerning puppies, adolescents, young/older adults, and seniors. This categorized age was employed only for descriptive purposes and continuous age was used in the other analyses. Owners were asked to report the dog’s exact weight in pounds, to the best of their knowledge. We converted the unit of weight from pounds to kilograms and classified them into five size groups: <10 kg, 10 to <20 kg, 20 to <30 kg, 30 to <40 kg, or 40+ kg. We used the converted weight as continuous for analysis results but as categorical in presenting the predictive pattern of disease history across continuous age.

### Statistical analysis

To understand the trend of disease history across age and size, we modeled lifetime prevalence of each disease category in three ways: 1) as a function of age and weight; 2) as a function of age, weight, and the age by weight interaction; and 3) as a function of age, weight, and the age by weight interaction, and adjusted for the dog’s sex, breed status, and census division. The response variable in the model is a count of the number of owner-reported “yes” responses to the question whether the dog has ever been diagnosed with condition(s) in a category regardless of the current status and the number of diagnoses in each category. We used continuous age and weight, and they were standardized by subtracting their means and dividing by their standard deviations. Dog’s sex was classified into four levels–neutered/intact male, or spayed/intact female–combining responses to the female vs. male question and whether the dog has been spayed or neutered. Breed status was analyzed as a binary variable (purebred vs. mixed-breed). For census division, we used the reported state of address where the dog resides and divided the information into nine census divisions that are adopted by the United States Census Bureau [19].

Poisson regression with robust standard error estimates [20] was used to estimate lifetime prevalence ratios (PR) and construct 95% confidence intervals (CI) of the PRs. A dispersion test was performed to check for over-dispersion in the model. Wald tests were used to test whether lifetime prevalence increased with age, and whether the size of the dog influenced either the lifetime prevalence of a reported condition in the disease category or its pattern across age. For ease of presentation we separated the result tables and figures into two sections: one for disease categories that tend to have a positive association with dog size, and one for disease categories for which lifetime prevalence is either lower with larger sizes or is not associated with dog size at all. This was based on the estimated PRs with increasing weight from the unadjusted model without the interaction term. In addition, we graphically illustrated the predicted lifetime prevalence of diagnoses within each disease category as a function of continuous age for each size class. These estimates were predicted using Poisson regression models fit with continuous age, categorical size, and the interaction of those two as predictors. The Poisson model uses the log of lifetime prevalence as a response and hence the relationships between age and lifetime prevalence appear curvilinear and provide a good match to the observed patterns. Though fitted models included dogs aged 15+ years, we produced predictions for age from 0 to 15 years, as dogs older than 15 were very rare, comprising only 3% of the study population. Given the large sample size of the study and continuous nature of the exposures we used p<0.01 to denote statistical significance. For interactions with p<0.01 we also visually inspected the figures for qualitative evidence of important interaction.

All statistical analyses were performed in R v4.1.2 [21]. The Poisson regression models were fit using the ‘glm’ function in the stats package. The dispersion test was performed using the ‘dispersiontest’ function in the AER package. The DAP is an open data project. These data are made available to the general public at dogagingproject.org/open_data_access [22].

## Results

There were 27,541 dog owners that completed the HLES and whose dogs are included in this report. The dogs ranged in age from puppies to very senior dogs, with a median age of 7 years (IQR 4 to 11). The dogs were equally distributed by sex (50% male) and purebred versus mixed-breed status (49% purebred). A total of 238 breeds were represented, and the frequency of each breed is provided in S1 Table. The respondents were distributed across the US, with the following percentages according to census divisions in decreasing order of representation: Pacific 24%, South Atlantic 18%, East North Central 15%, Mountain 10%, Mid-Atlantic 9%, West South Central 8%, New England 6%, West North Central 6%, East South Central 3%. Owners most commonly described the locations where they lived with their dogs as suburban (62%), with 17% residing in urban locations and 21% in rural locations.

We present the frequency and proportion of owner-reported lifetime prevalence of categorical diagnoses by age categories in Table 1, and by weight categories in Table 2. Disease categories are listed in descending order of overall frequency.

**Table 1:** Proportion with disease history by age category.

Disease	Puppy (<1yr)	Adolescent (1 to <3yr)	Young Adult (3 to <7yr)	Older Adult (7 to <11yr)	Senior (≥11yr)	Overall
	N = 591	N = 4622	N = 8249	N = 7666	N = 6413	N = 27541
Skin	40 (7%)	796 (17%)	2181 (26%)	2556 (33%)	2342 (37%)	7915 (29%)
Infection/Parasites	147 (25%)	1269 (27%)	2291 (28%)	2009 (26%)	1623 (25%)	7339 (27%)
Bone/Orthopedic	7 (1%)	239 (5%)	849 (10%)	1699 (22%)	2493 (39%)	5287 (19%)
Gastrointestinal	39 (7%)	488 (11%)	1059 (13%)	1158 (15%)	1170 (18%)	3914 (14%)
Ocular	31 (5%)	283 (6%)	586 (7%)	906 (12%)	1819 (28%)	3625 (13%)
Ear/Nose/Throat	19 (3%)	302 (7%)	745 (9%)	916 (12%)	1587 (25%)	3569 (13%)
Kidney/Urinary	14 (2%)	153 (3%)	406 (5%)	591 (8%)	957 (15%)	2121 (8%)
Cancer/Tumors	1 (<1%)	21 (<1%)	177 (2%)	586 (8%)	966 (15%)	1751 (6%)
Cardiac	3 (<1%)	45 (<1%)	167 (2%)	440 (6%)	912 (14%)	1567 (6%)
Brain/Neurologic	2 (<1%)	25 (<1%)	188 (2%)	359 (5%)	750 (12%)	1324 (5%)
Liver/Pancreas	2 (<1%)	24 (<1%)	125 (2%)	277 (4%)	542 (8%)	970 (4%)
Respiratory	4 (<1%)	53 (1%)	132 (2%)	242 (3%)	519 (8%)	950 (3%)
Endocrine	0 (<1%)	8 (<1%)	77 (<1%)	310 (4%)	518 (8%)	913 (3%)

Source: Data from the Dog Aging Project for N = 27,541 dogs included in the 2020 data release.

**Table 2:** Proportion with disease history by weight category.

Disease	<10kg	10 to <20kg	20 to <30kg	30 to <40kg	> = 40kg	Overall
	N = 6207	N = 5613	N = 8219	N = 5167	N = 2335	N = 27541
Skin	1628 (26%)	1531 (27%)	2369 (29%)	1621 (31%)	766 (33%)	7915 (29%)
Infection/Parasites	1186 (19%)	1593 (28%)	2441 (30%)	1478 (29%)	641 (27%)	7339 (27%)
Bone/Orthopedic	1187 (19%)	931 (17%)	1461 (18%)	1181 (23%)	527 (23%)	5287 (19%)
Gastrointestinal	892 (14%)	786 (14%)	1119 (14%)	749 (14%)	368 (16%)	3914 (14%)
Ocular	1079 (17%)	800 (14%)	934 (11%)	571 (11%)	241 (10%)	3625 (13%)
Ear/Nose/Throat	827 (13%)	713 (13%)	915 (11%)	752 (15%)	362 (16%)	3569 (13%)
Kidney/Urinary	530 (9%)	452 (8%)	639 (8%)	369 (7%)	131 (6%)	2121 (8%)
Cancer/Tumors	273 (4%)	327 (6%)	565 (7%)	428 (8%)	158 (7%)	1751 (6%)
Cardiac	671 (11%)	362 (6%)	301 (4%)	177 (3%)	56 (2%)	1567 (6%)
Brain/Neurologic	362 (6%)	278 (5%)	346 (4%)	235 (5%)	103 (4%)	1324 (5%)
Liver/Pancreas	393 (6%)	209 (4%)	195 (2%)	130 (3%)	43 (2%)	970 (4%)
Respiratory	385 (6%)	169 (3%)	207 (3%)	124 (2%)	65 (3%)	950 (3%)
Endocrine	194 (3%)	190 (3%)	264 (3%)	176 (3%)	89 (4%)	913 (3%)

Source: Data from the Dog Aging Project for N = 27,541 dogs included in the 2020 data release.

We now provide results for each disease category individually, with the categories that were positively associated with dog size discussed first, followed by those that were either negatively or not associated with dog size. From the dispersion test, we found no significant evidence of over-dispersion in the fitted models for every condition. Adjustment for sex, breed status and geographic region did not have any notable impact on the associations, and this was true for all disease categories we studied. In the Supporting Information (S1 and S2 Appendices), we presented the associations between weight and each disease prevalence across age, using subsets of all purebred and all mixed breed dogs.

### Skin conditions

**Fig 1:**
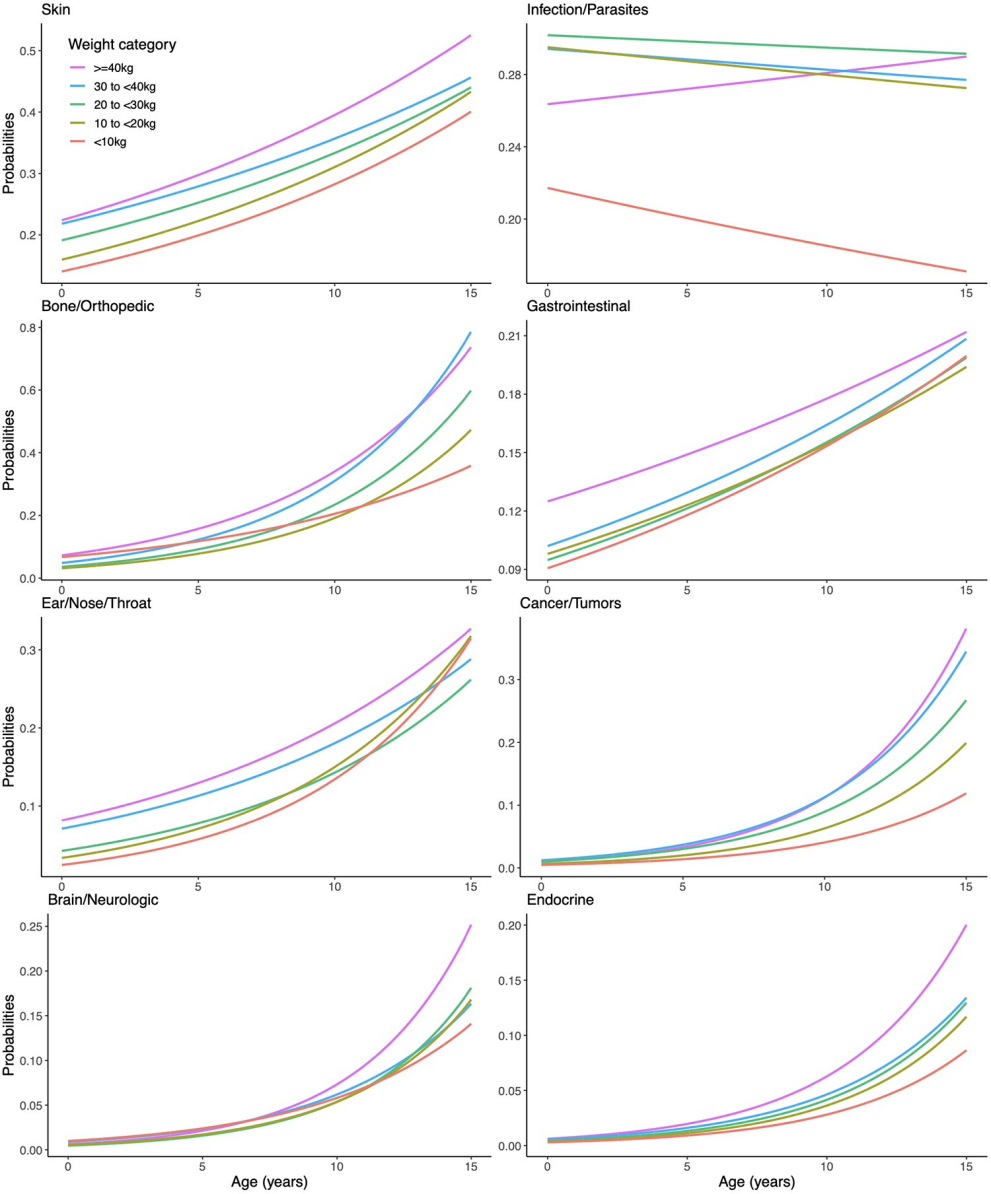
Results from Model 2 with continuous age by weight category (Part 1: Conditions positively associated with weight from Model 1). Data from the Dog Aging Project for N = 27,541 dogs included in the 2020 data release.

A total of 7915 (28.7%) dog owners reported that their dogs had a history of conditions within the skin disease category. The proportion of dogs with a reported history of skin conditions increased steadily with age, ranging from 7% in puppies to 37% in senior dogs (Table 1). History of skin disease was reported less for smaller vs. larger dogs, ranging from 26% in dogs <10 kg to 33% in dogs over 40 kg (Table 2). In the Poisson regression model with the main effects of age and weight, each SD increment of age (4 years) was associated with a 29% greater relative lifetime prevalence of a reported history of skin conditions. Lifetime prevalence also increased with the owner-reported weight of the dog, with each SD increment (13 kgs) associated with 12% higher relative lifetime prevalence of skin condition history (Table 3). In Fig 1, we illustrate these patterns using a categorical representation of dog size. We see strongly increasing trends over age that are similar across size classes. Toy dogs have the lowest rates across age, and a history of skin conditions is reported progressively more often for each successively larger size category regardless of age at the time of the survey. Older dogs were reported to have skin conditions more often by a similar relative margin across different size classes, which was in alignment with the results in Table 3 that there was no significant interaction between age and weight.

**Table 3:** Association of age and weight with lifetime prevalence (Part 1: Conditions positively associated with weight from Model 1).

		Model 1^a^			Model 2^b^			Model 3^c^		
Condition	Characteristic	PR	95% CI	p-value	PR	95% CI	p-value	PR	95% CI	p-value	
Skin	Age	1.29	(1.27, 1.31)	<0.001	1.29	(1.27, 1.31)	<0.001	1.27	(1.25, 1.30)	<0.001	
	Weight	1.12	(1.10, 1.14)	<0.001	1.12	(1.10, 1.14)	<0.001	1.13	(1.11, 1.15)	<0.001	
	Age*Weight	-	-	-	0.98	(0.96, 1.00)	0.02	0.98	(0.96, 0.99)	<0.01	
Infection/Parasites	Age	0.97	(0.95, 0.99)	<0.01	0.97	(0.96, 0.99)	0.01	0.96	(0.94, 0.98)	<0.001	
	Weight	1.09	(1.07, 1.11)	<0.001	1.10	(1.08, 1.12)	<0.001	1.11	(1.09, 1.13)	<0.001	
	Age*Weight	-	-	-	1.04	(1.02, 1.06)	<0.001	1.04	(1.02, 1.06)	<0.001	
Bone/Orthopedic	Age	2.00	(1.95, 2.04)	<0.001	2.00	(1.96, 2.05)	<0.001	1.98	(1.93, 2.03)	<0.001	
	Weight	1.22	(1.19, 1.25)	<0.001	1.17	(1.13, 1.20)	<0.001	1.17	(1.13, 1.20)	<0.001	
	Age*Weight	-	-	-	1.08	(1.05, 1.11)	<0.001	1.08	(1.05, 1.11)	<0.001	
Gastrointestinal	Age	1.22	(1.19, 1.26)	<0.001	1.22	(1.19, 1.26)	<0.001	1.21	(1.18, 1.25)	<0.001	
	Weight	1.04	(1.01, 1.07)	0.01	1.04	(1.01, 1.07)	0.01	1.03	(1.00, 1.06)	0.06	
	Age*Weight	-	-	-	0.98	(0.96, 1.01)	0.24	0.99	(0.96, 1.02)	0.42	
Ear/Nose/Throat	Age	1.76	(1.70, 1.82)	<0.001	1.74	(1.68, 1.79)	<0.001	1.73	(1.68, 1.79)	<0.001	
	Weight	1.16	(1.12, 1.19)	<0.001	1.21	(1.17, 1.24)	<0.001	1.19	(1.16, 1.23)	<0.001	
	Age*Weight	-	-	-	0.89	(0.87, 0.92)	<0.001	0.90	(0.87, 0.92)	<0.001	
Cancer/Tumors	Age	2.56	(2.45, 2.67)	<0.001	2.54	(2.43, 2.66)	<0.001	2.52	(2.41, 2.64)	<0.001	
	Weight	1.40	(1.35, 1.45)	<0.001	1.33	(1.27, 1.39)	<0.001	1.35	(1.28, 1.41)	<0.001	
	Age*Weight	-	-	-	1.07	(1.03, 1.11)	<0.001	1.07	(1.02, 1.11)	<0.01	
Brain/Neurologic	Age	2.46	(2.33, 2.59)	<0.001	2.48	(2.35, 2.62)	<0.001	2.49	(2.35, 2.63)	<0.001	
	Weight	1.08	(1.02, 1.15)	<0.01	1.04	(0.97, 1.12)	0.30	1.01	(0.94, 1.09)	0.70	
	Age*Weight	-	-	-	1.06	(1.00, 1.12)	0.05	1.07	(1.02, 1.13)	0.01	
Endocrine	Age	2.60	(2.46, 2.75)	<0.001	2.60	(2.46, 2.75)	<0.001	2.59	(2.45, 2.74)	<0.001	
	Weight	1.27	(1.19, 1.35)	<0.001	1.26	(1.18, 1.35)	<0.001	1.24	(1.16, 1.33)	<0.001	
	Age*Weight	-	-	-	1.01	(0.96, 1.06)	0.74	1.02	(0.97, 1.07)	0.52	

Source: Data from the Dog Aging Project for N = 27,541 dogs included in the 2020 data release.

Note: Age (years) and Weight (kg) are standardized by subtracting their means, 7 and 23, and dividing by their standard deviations, 4 and 13, respectively.

^a^A model with the main effects of age and weight.

^b^A model with the main effects of age, weight, and the interaction.

^c^A model with the main effects of variables in Model 2 plus adjusted for sex, purebred/mixed-breed, and geographic region.

### Infectious diseases

A total of 7339 (26.6%) dog owners reported that their dogs had a history of conditions within the infectious disease category. The proportion of dogs with a reported history of infectious diseases followed a unique pattern relative to all other conditions studied. The lifetime prevalence among puppies was 25%, and this did not increase across other age groups ranging between 25–28% with no particular pattern (Table 1). The smallest dogs <10 kg had the lowest reported history of infectious diseases at 19%, but all other size classes were similar to each other, with 27–30% lifetime prevalence (Table 2). These patterns are most clearly illustrated in Fig 1, where the pattern across age groups is flat, and the curve for the smallest dogs is lowest.

### Orthopedic conditions

A total of 5287 (19.2%) dog owners reported that their dogs had a history of conditions within the orthopedic disease category. The proportion of dogs reported to have a history of orthopedic conditions increased slightly with size (Table 2). On average, the lifetime prevalence of a reported history of orthopedic conditions doubled with every SD increment in age, but as indicated by the interaction term, the lifetime prevalence across age increased much more sharply as the size of the dog increased. This is illustrated in Fig 1, where for puppies and adolescents (<3 years) there is very little difference in lifetime prevalence by size class, but for older adult to senior (7+ years) dogs, the larger dogs have a much greater lifetime prevalence of a history of orthopedic conditions.

### Gastrointestinal conditions

A total of 3914 (14.2%) dog owners reported that their dogs had a history of conditions within the gastrointestinal disease category. The reported proportion of dogs with a history of gastrointestinal conditions increased with age from 7% in puppies to 18% among senior dogs but only slightly with size from 14% in dogs <10 kg to 16% in dogs over 40 kg. Each SD increment in dog age was associated with a 22% higher relative lifetime prevalence, while each SD increment in dog weight was associated with 4% greater relative lifetime prevalence (Table 3). In Fig 1 we see that all size groups increase steadily with age. Dogs <30 kg are all similar, dogs between 30–40 kg have somewhat higher lifetime prevalence, and dogs over 40 kg have notably higher lifetime prevalence across all ages.

### Ear, nose, and throat conditions

A total of 3569 (13%) dog owners reported that their dogs had a history of conditions within the ear, nose, and throat (ENT) disease category. Reported history of ENT conditions increased from 3% among puppies to 25% among senior dogs (Table 1). Trends across size were slight, ranging from 13% in the smallest dogs (<10kg) to 16% in the largest dogs (≥40 kg) (Table 2). Controlling for the weight of the dog, on average the relative lifetime prevalence of ENT conditions was 76% higher for each SD increment in age. However, there was a significant interaction between size and weight (Table 3). Specifically, for larger dogs the trend across age was flatter. Puppies to young adults (<7 years) had higher lifetime prevalence of ENT history for larger dogs, but for seniors (11+ years), small dogs (<20 kg) had caught up to the largest dogs. This is illustrated in Fig 1.

### Cancer/tumors

A total of 1751 (6.4%) dog owners reported that their dogs had a history of conditions within the cancer/tumor category. The proportion of dogs for whom a history of cancer was reported increased sharply with age, ranging from <1% in puppies to 15% in senior dogs (Table 1). Lifetime prevalence by size ranged from 4% in dogs <10 kg, to between 6–8% among dogs over 10 kg (Table 2). The increasing pattern across age was much more pronounced in larger dogs. For dogs of the same size, each SD increment in dog age was associated with a two-and-a-half-fold increase in the lifetime prevalence of cancer history. For each additional SD increment in size, the age trend increased by 7% (Table 3). In Fig 1 we see this illustrated graphically, where for puppies and adolescents (<3 years), lifetime prevalence is low for all sizes, but for seniors (11+ years) the lifetime prevalence has risen rapidly, especially for dogs over 30 kg.

### Neurologic conditions

A total of 1324 (4.8%) dog owners reported that their dogs had a history of conditions within the neurologic disease category. The proportion of dogs with a history of neurologic conditions increased from <1% in puppies and adolescent dogs to 12% in senior dogs (Table 1). On average there was no apparent trend across sizes, though the largest dogs had a much steeper increasing pattern across age than dogs <40 kg (Table 2). In Fig 1, before older adulthood the lifetime prevalence of neurologic conditions was low, and similar across size classes. In older adult and senior dogs the lifetime prevalence increased in a similar way for dogs <40 kg, and more steeply for dogs over 40 kg.

### Endocrine conditions

A total of 913 (3.3%) dog owners reported that their dogs had a history of conditions within the endocrine disease category. The lifetime prevalence of a reported history of endocrine conditions was <1% through young adulthood, 4% for older adult dogs, and 8% for senior dogs (Table 1). On average there was not a strong size pattern with lifetime prevalence ranging between 3–4% (Table 2). However, controlling for age the influence of size on lifetime prevalence was apparent (Table 3). The increasing pattern of endocrine condition history across age was similar for all size classes. These patterns are illustrated in Fig 1, where the lifetime prevalence of reported history of endocrine conditions increases over time at a similar rate for each size class, but the larger the dog the higher the estimated lifetime prevalence curve.

### Ocular conditions

**Fig 2:**
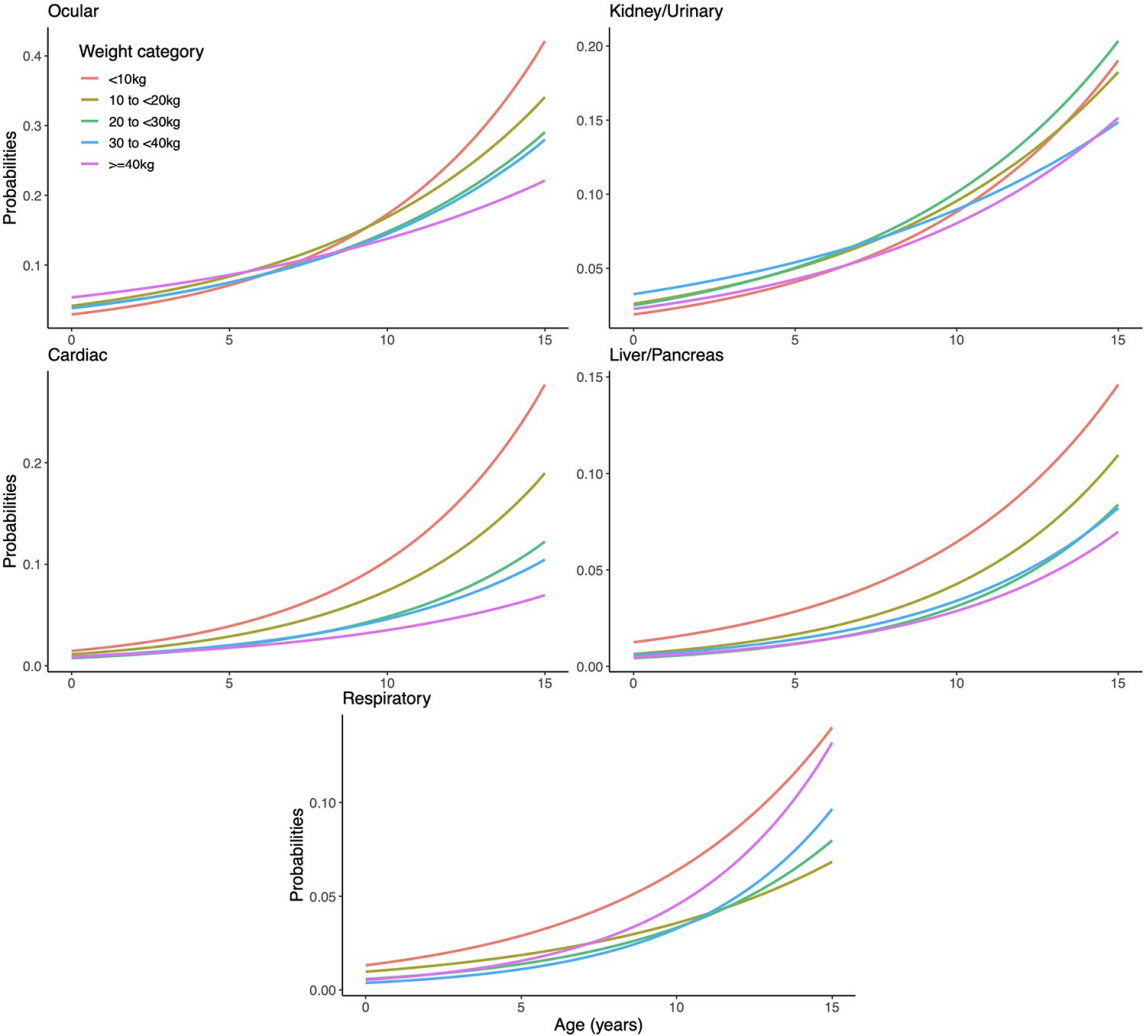
Results from Model 2 with continuous age by weight category (Part 2: Conditions negatively associated or not associated with weight from Model 1). Data from the Dog Aging Project for N = 27,541 dogs included in the 2020 data release.

A total of 3625 (13.2%) dog owners reported that their dogs had a history of conditions within the ocular disease category. A reported history of ocular conditions increased steadily with age group, from 5% in puppies to 28% among senior dogs (Table 1). In contrast, the proportion with a positive history was lower among larger dogs, ranging from 17% in the smallest dogs to just 10% in the dogs over 40 kg (Table 2). This was reflected in the models where the significant interaction between age and weight is seen as a steeper slope in smaller dogs (Table 4). Graphically we can see in Fig 2 that below adulthood the size categories have roughly the same lifetime prevalence but then the smaller dogs increase much more quickly in reported history of ocular conditions.

**Table 4:** Association of age and weight with lifetime prevalence (Part 2: Conditions negatively associated or not associated with weight from Model 1).

		Model 1^a^			Model 2^b^			Model 3^c^		
Condition	Characteristic	PR	95% CI	p-value	PR	95% CI	p-value	PR	95% CI	p-value	
Ocular	Age	1.86	(1.81, 1.92)	<0.001	1.80	(1.74, 1.86)	<0.001	1.81	(1.75, 1.87)	<0.001	
	Weight	0.93	(0.90, 0.96)	<0.001	0.98	(0.94, 1.02)	0.27	0.96	(0.93, 1.00)	0.05	
	Age*Weight	-	-	-	0.89	(0.87, 0.92)	<0.001	0.91	(0.88, 0.94)	<0.001	
Kidney/Urinary	Age	1.76	(1.69, 1.83)	<0.001	1.75	(1.68, 1.82)	<0.001	1.72	(1.65, 1.79)	<0.001	
	Weight	1.01	(0.97, 1.05)	0.77	1.02	(0.98, 1.07)	0.32	1.07	(1.02, 1.12)	<0.01	
	Age*Weight	-	-	-	0.96	(0.93, 1.00)	0.06	0.94	(0.91, 0.98)	<0.01	
Cardiac	Age	2.19	(2.10, 2.29)	<0.001	2.10	(1.98, 2.21)	<0.001	2.11	(2.00, 2.23)	<0.001	
	Weight	0.67	(0.63, 0.71)	<0.001	0.71	(0.66, 0.77)	<0.001	0.70	(0.65, 0.75)	<0.001	
	Age*Weight	-	-	-	0.92	(0.87, 0.97)	<0.01	0.93	(0.88, 0.99)	0.02	
Liver/Pancreas	Age	2.12	(2.00, 2.24)	<0.001	2.12	(1.99, 2.26)	<0.001	2.12	(1.98, 2.26)	<0.001	
	Weight	0.75	(0.69, 0.81)	<0.001	0.75	(0.68, 0.82)	<0.001	0.75	(0.68, 0.82)	<0.001	
	Age*Weight	-	-	-	1.01	(0.94, 1.08)	0.85	1.02	(0.95, 1.09)	0.67	
Respiratory	Age	2.01	(1.89, 2.14)	<0.001	2.04	(1.90, 2.20)	<0.001	2.06	(1.91, 2.21)	<0.001	
	Weight	0.80	(0.74, 0.87)	<0.001	0.78	(0.71, 0.86)	<0.001	0.76	(0.69, 0.84)	<0.001	
	Age*Weight	-	-	-	1.04	(0.96, 1.13)	0.32	1.06	(0.98, 1.15)	0.13	

Source: Data from the Dog Aging Project for N = 27,541 dogs included in the 2020 data release.

Note: Age (years) and Weight (kg) are standardized by subtracting their means, 7 and 23, and dividing by their standard deviations, 4 and 13, respectively.

^a^A model with the main effects of age and weight.

^b^A model with the main effects of age, weight, and the interaction.

^c^A model with the main effects of variables in Model 2 plus adjusted for sex, purebred/mixed-breed, and geographic region.

### Kidney or urinary conditions

A total of 2122 (7.7%) dog owners reported that their dogs had a history of conditions within the kidney or urinary disease category. The proportion of dogs with reported history of kidney or urinary conditions increased steadily with age, from 2% in puppies to 15% in senior dogs (Table 1). Across size categories differences were not dramatic, but the lifetime prevalence was highest in the smallest dogs at 9% in dogs <10 kg and lowest in largest dogs at 6% in dogs ≥40 kg (Table 2). Differences between size classes were most apparent in older adults and seniors, as evidenced by the significant age by weight interaction, and the graphical representation in Fig 2.

### Cardiac conditions

A total of 1567 (5.7%) dog owners reported that their dogs had a history of conditions within the cardiac disease category. The proportion of dogs with a reported history of cardiac conditions exhibited strong trends across both age and dog size. Among puppies and adolescents the proportion was <1%, increasing to 14% among senior dogs (Table 1). Dog weight was inversely associated, with dogs <10 kg reporting an 11% lifetime prevalence of a history of cardiac conditions, declining to only 2% among the largest dogs over 40 kg (Table 2). These trends were highly significant, with each SD increment in age associated with over a doubling of lifetime prevalence, and each SD increment in size reducing lifetime prevalence by a third (Table 4). Additionally, we observed a significant interaction where the lifetime prevalence was not only higher for smaller dogs but was associated with a significantly steeper increase in lifetime prevalence across age groups.

### Liver or pancreas conditions

A total of 970 (3.5%) dog-owners reported that their dogs had a history of conditions within the liver or pancreas disease category. A reported history of liver or pancreas conditions was more common among older dogs ranging from <1% in puppies and adolescent dogs to 8% in senior dogs (Table 1). In contrast the proportion was lower among larger dogs, ranging from 6% in the smallest size category down to 2% among larger dogs (Table 2). Each SD increment in age more than doubled the lifetime prevalence of a reported history of liver/pancreas conditions, while each SD increment in size reduced the lifetime prevalence by approximately one quarter (Table 4). The smallest dog category (<10 kg) in particular had a notably higher lifetime prevalence of liver condition history in Fig 2.

### Respiratory conditions

A total of 950 (3.4%) dog owners reported that their dogs had a history of conditions within the respiratory disease category. The proportion of dogs with a reported history of respiratory conditions went up with increased age, from <1% in puppies to 8% in senior dogs (Table 1). The proportion decreased with increasing size, from 6% in the smallest dogs to 3% in the largest dogs (Table 2). For every 1 SD increment in age the lifetime prevalence of a reported history of respiratory conditions doubled, while for every 1 SD increment in weight the relative lifetime prevalence was 20% lower (Table 4). These patterns are illustrated in the final panel of Fig 2.

## Discussion

As found in other retrospective studies of the domestic dog [1, 11, 23–27], we found that owner-reported diagnoses (grouped by organ system or pathophysiologic process) disproportionately affect dogs of different sizes. Some conditions were reported less commonly for larger dogs and these included ocular, cardiac, liver/pancreas, and respiratory conditions. The proportion reporting a history of urinary conditions did not vary by weight. Many conditions were reported more commonly with increasing weight category, including skin, orthopedic, gastrointestinal, ENT, cancer, neurologic, and endocrine conditions. The infectious disease category showed a distinct pattern that the smallest dogs (<10 kg) had much lower lifetime prevalence than the other categories and there were no increasing patterns across age. With the exception of infectious diseases, the proportion of dogs with a reported history of each disease category increased with increasing age group, as expected. For different disease categories, size had various effects on the relative lifetime prevalence of reporting a history of disease as well as on the slope of increasing lifetime prevalence across age.

Higher growth rates in larger dogs have been implicated in increased oxidative damage during early life that may predispose to certain diseases such as skin diseases, orthopedic conditions, cancer, and cardiac disorders [28]. Skin is a common site for evidence of oxidative damage; increased production of free radicals including reactive oxygen species can overwhelm the body’s mechanisms for reducing their damaging effects and manifest as skin lesions and other pathology [29]. In mouse models, increased oxidative stress induced by exposure to diisodecyl phthalate exacerbated symptoms of allergic dermatitis [30]. A retrospective study of 721 dogs by Dreschel [31] measuring associations between behavioral health and physical health found that dogs with severe fear and separation anxiety disorders had both significantly higher lifetime prevalence and severity of skin diseases [31]. The author suggests that these associations may be due to physiological stress responses in dogs with behavioral disorders and resulting changes in hormone regulation, immunity, and disease risk.

Faster growth rates and larger body size have also been associated with increased risk for orthopedic diseases [6]. Growth patterns in larger dog breeds involve rapid weight gain throughout the period of skeletal development and maturity, which has been linked to increased risk of developmental musculoskeletal and orthopedic diseases including hip dysplasia, osteoarthritis, and osteochondrosis [6, 32, 33]. In our cohort, osteoarthritis was spread similarly among dogs with body weight greater than ten kilograms. The morbidity associated with osteoarthritis may vary. Orthopedic pain from any of these diagnoses can significantly reduce quality of life (QOL) [34]. Poor QOL has been identified as one of the most influential factors for owners choosing to euthanize their dogs and thereby shorten their lifespan to prevent pain and distress [35]. Most medications commonly used to alleviate pain from osteoarthritis are dosed by weight and are thus more likely to be cost-prohibitive for owners with larger dogs; such factors may also lead to differential effects of osteoarthritis by size on QOL and decisions about euthanasia. Additionally, mobility problems due to orthopedic disease are more easily accommodated in dogs of smaller size, as owners are more likely to be capable of lifting or carrying smaller dogs and/or assisting their movement with slings, harnesses, and other devices. It is important to note that the analyses presented here included size only, and did not analyze subpopulations of specific breeds. Because members of a breed are similar in size, it is possible that common breeds within this cohort contributed disproportionately to the findings for a given size.

Across all body sizes, several retrospective studies of large cohorts of companion dogs in North America and Europe have consistently found that cancer [1, 24, 26, 27, 35, 36] is the most common cause of mortality in pet dogs. Multiple studies that have considered body size in relation to causes of morbidity and mortality have reported that cancer diagnoses are more common in larger dogs than smaller dogs [1, 37–39]. Several theories about the contributing factors to reduced longevity in larger dogs are consistent with increased risk of cancer in larger dogs including correlations between increased serum IGF-1 levels in larger dogs and downstream effects on growth, oxidative damage, and cancer risk [6, 37, 40, 41]. Artificial selection to produce the extreme variation in growth rates among breeds has resulted in wide variation in serum IGF-1 levels before and after skeletal maturity. For example, Great Danes gained weight at 17 times the rate of miniature poodles in a study following the first 21 weeks of life [42]. Correspondingly higher levels of mean basal IGF-1 detected in the plasma of Great Danes compared to miniature poodles from age 13 weeks to 27 weeks in a related study suggested that Great Danes may enter a period of physiologic gigantism postnatally [43]. Plasma levels of IGF-1 remain higher in larger breed dogs compared to smaller breed dogs past maturity, and this inverse correlation between IGF-1 levels and longevity is consistent with patterns found in many other species [6].

Studies investigating the relationship between average breed growth rates and prevalence of life-limiting disorders linked to oxidative damage have revealed correlations between breed size and risk of developing cardiac pathology. Increased risk of cardiac disease and associated morbidity and mortality have been reported at both extremes of the body size spectrum in dogs, though the prevalence of specific cardiac pathophysiologies varies widely from small dogs to giant breed dogs [1, 26, 27]. Artificial selection for extreme size (both small and giant) may have contributed to the distribution of cardiac disease risk in dogs, as many genes associated with body size in dogs also contribute to cardiac development and structure [44]. Telomere length in peripheral blood mononuclear cells was positively correlated with lifespan and inversely correlated with risk of mortality due to cardiac disease in a study of 15 breeds of dog, with the shortest age-adjusted telomere length in the Great Dane [45]. In our cohort, reported lifetime prevalence of cardiac disease increases more rapidly with age in smaller dogs but the associated contributions of these diseases to mortality is unknown.

In our cohort, larger dogs were also more likely to have diagnoses in the neurological category. The most commonly reported specific condition was seizures (including epilepsy) and it was distributed fairly evenly across body sizes (Table 10 in S3 Appendix). Fleming *et al*. [1] found that larger dogs were relatively spared from death due to neurological diseases. Our results are not inconsistent with these findings as our study does not address cause of death but rather disease history, and many dogs with seizures do not die as a direct result of their neurological disease; the majority of dogs with epilepsy (the most common cause of recurrent seizures in dogs) can be treated successfully with conventional drugs [46] and, similarly, the majority of companion dogs with epilepsy die of a cause not directly related to epilepsy [47]. Additionally, neurological diseases found more commonly in smaller dogs (e.g., intervertebral disc disease) tend to present later in life than epilepsy such that neurological disease in smaller dogs would be less frequent in our cohort than in cohorts examined retrospectively for causes of mortality. This observation aligns with the pattern that the risk of certain diseases in dogs can be associated with their size in various ways, either by increasing, decreasing, or being independent of their size.

Previously, Fleming *et al*. [1] reported that larger breeds are spared from death by endocrine disease. In our cohort, the reported lifetime prevalence of a diagnosis in the endocrine category was more likely in larger dogs. This contrast between previous reported mortality versus lifetime prevalence in our study may be driven almost entirely by the most commonly reported specific endocrine disorder, hypothyroidism (Table 13 in S3 Appendix). Hypothyroidism was more prevalent in larger dogs, and this diagnosis alone was responsible for most of the trend by size for the endocrine category. While untreated hypothyroidism can cause significant morbidity and even mortality, it is relatively easy and inexpensive to treat in dogs [48, 49]. Cushing’s disease and diabetes mellitus, two frequently diagnosed diseases that are more prevalent in smaller dogs in our cohort, carry greater morbidity and mortality risk than hypothyroidism if untreated, and treatment places a greater financial burden for the owner compared to typical treatments for hypothyroidism [50–53]. It is noteworthy that Fleming *et al*. [1] used data from dogs seen in referral institutions while the dogs in our study were recruited directly from the community. It is therefore likely that referral bias [54] could also explain the difference in these results, as treatment of hypothyroidism is usually straightforward and does not require referral [55].

For the ENT category, the most commonly reported diagnosis, ear infection, was drastically skewed toward larger dogs. Ear infections in dogs are commonly associated with allergic skin disease and food allergies and thus increased lifetime prevalence in larger dogs would be consistent with the aforementioned theories about increased oxidative damage and skin manifestations. While ear infections can sometimes be easily treated, chronic/recurrent ear infections are also common and can drastically affect QOL [56–59]. Recurrent ear infections can be expensive to monitor and treat, with surgery to remove the entire ear canal(s) as the only curative option for some dogs. Because significant financial resources may be necessary to avoid negative impacts on QOL due to ear infections, cost may be a factor guiding euthanasia decisions and could thus influence lifespan [59, 60]. The next most frequent diagnoses in the ENT category were hearing loss and deafness, which both have frequencies skewed toward smaller dogs. In general, pet dogs do not receive treatment for hearing loss or deafness, nor do they receive adaptive devices such as hearing aids and thus financial limitations of the owner do not tend to change the prognosis for the patient with chronic ear infections. Hearing loss is typically not associated with pain, and dogs are often capable of maintaining a good QOL despite partial or complete deafness.

Diagnoses within the respiratory category were more common overall among smaller dogs. The most common specific diagnoses were chronic cough and tracheal collapse, both of which are known to be more common among smaller dogs [61, 62]. Additionally, components of the brachycephalic syndrome (hypoplastic trachea, redundant soft palate and stenotic nares) were among the top ten diagnoses in the respiratory category and were more often noted among smaller dogs [63]. Currently a number of small breeds with extreme brachycephalic phenotype, such as French Bulldogs and Pugs, are popular in the US and consequently within the DAP Pack [63, 64].

Our study has several strengths and limitations that should be noted. Strengths include the large sample size of this study, which allows us to estimate patterns with high power across the whole age and size spectrum. Additionally, we have a very diverse sample of dogs distributed across the entire United States. Since the sample is not veterinary-hospital or clinic-based it may be more representative of the general population of dogs. Conversely, while our observations can suggest which conditions manifest differently across age and size, they do not prove any causal relationships due to the cross-sectional nature of the analysis. Over time, longitudinal data will be collected on these dogs, and we will be able to examine disease incidence. In addition, recall bias may occur when owners fill out the survey. It is possible that owners may not remember past events at all or incorrectly at the time of the survey. Response bias may also occur when owners who lack understanding of disease categories provide inaccurate answers. Additionally, we acknowledge a low level of potential errors in reporting the dog’s exact weight in pounds in the survey. There is also potential for measurement error because weight is not a precise measure of a dog’s size and can be affected by factors such as the normal or overweight status. Future studies will compare HLES data to Veterinary Electronic Medical Records data to measure the accuracy of owner-reported responses and confirm the trends seen in HLES. Lifetime prevalence of conditions was expected to go up regardless of an increasing age-specific prevalence because it was calculated in a cumulative way. In addition, the lifetime prevalence for conditions which rapidly lead to mortality will be underestimated in this sample. Finally, the sample is not random but self-selected so that data are subject to self-selection bias. Owners who are more exposed to this survey and who tend to participate in surveys are more likely to nominate their dogs and complete the survey, possibly leading to a biased sample. The restriction of allowing only one dog per household to enroll in the DAP could also introduce bias, favoring either healthy pets or those with notable medical histories. The DAP endeavors to create a representative sample of the companion canine population in the US. However, computer access is a necessary condition for survey completion, as well as the ability to complete the surveys in English.

In our analyses, we did not control for individual breeds for several reasons. First, a significant portion of our study population consisted of mixed breed dogs, and previous studies have demonstrated the unreliability of owner-reported ancestry for such dogs [65]. In addition, adjusting for individual breed would generate an assessment of the impact of body condition (overweight or underweight) and skeletal variation within each breed on lifetime disease prevalence, which is not the focus of our study. Furthermore, in the absence of a specific understanding of the genetic origins of a given disease, studies of breed and disease risk may lead to inaccurate impressions of the frequency and magnitude of breed-based risk. Studies have also shown that whether certain genetic disorders are more common among purebred vs. mixed breed dogs depends on the condition [14]. For these reasons, we instead conducted separate analyses using subsets of all purebred and all mixed breed dogs and compared the results in the Supporting Information (S1 and S2 Appendices). Furthermore, we compared the results of the association of age and weight with lifetime prevalence from the entire set to those from a sample excluding the 10 most common breeds, one breed at a time. In this analysis, we observed only 5 among 1,040 cases where the significance of the association shifted from significant to non-significant. Future studies will examine the genetic architecture of diseases in this specific population.

In this study we have quantified the owner-reported lifetime prevalence of a history of conditions within several different disease categories as a function of dog age and size, with and without adjustment for sex, geographic location, and purebred versus mixed-breed status. These are lifetime disease prevalence data rather than cause-of-death data. However, these results nonetheless provide insights into the disease categories that may contribute to reduced lifespan in larger dogs and suggest multiple avenues for further exploration. More focused efforts to look at individual conditions within categories may yield additional insights. Within and across categories, the co-occurrence of different disease subtypes may also be an important factor to evaluate. Of course, as prospective data become available the longitudinal associations of these conditions with subsequent morbidity and mortality will be evaluated.

## Supporting information

S1 Table Owner-reported breed frequency. (XLSX)

S1 Appendix Analysis using subsets of all purebred dogs. (PDF)

S2 Appendix Analysis using subsets of all mixed breed dogs. (PDF)

S3 Appendix Summary tables of proportion with each disease/disorder history by weight category. (XLSX)
