# Supplementary material for: Dog size and patterns of disease history across the canine age spectrum: Results from the Dog Aging Project
Source: PLoS One. 2024 Jan 17;19(1):e0295840. doi: 10.1371/journal.pone.0295840 (PMC10793924; doi:10.1371/journal.pone.0295840)
Supplement: S1 Appendix — (PDF) [file pone.0295840.s002.pdf]

Analysis using subsets of all purebred dogs

Table 1: Proportion with Disease History by Age

	Puppy (<1yr)	Adolescent (1 to <3yr)	Young Adult (3 to <7yr)	Older Adult (7 to <11yr)	Senior (>=11yr)	Overall
Disease	N=339	N=2483	N=4018	N=3686	N=3092	N=13618
Skin	21 (6%)	416 (17%)	1057 (26%)	1198 (33%)	1117 (36%)	3809 (28%)
Infection/Parasites	76 (22%)	618 (25%)	1034 (26%)	906 (25%)	705 (23%)	3339 (25%)
Bone/Orthopedic	3 (<1%)	132 (5%)	434 (11%)	862 (23%)	1239 (40%)	2670 (20%)
Gastrointestinal	21 (6%)	282 (11%)	558 (14%)	578 (16%)	597 (19%)	2036 (15%)
Ocular	13 (4%)	154 (6%)	349 (9%)	519 (14%)	974 (32%)	2009 (15%)
Ear/Nose/Throat	11 (3%)	176 (7%)	415 (10%)	494 (13%)	828 (27%)	1924 (14%)
Kidney/Urinary	12 (4%)	81 (3%)	217 (5%)	329 (9%)	481 (16%)	1120 (8%)
Cancer/Tumors	1 (<1%)	13 (<1%)	87 (2%)	306 (8%)	464 (15%)	871 (6%)
Cardiac	0 (<1%)	28 (1%)	96 (2%)	247 (7%)	463 (15%)	834 (6%)
Brain/Neurologic	0 (<1%)	11 (<1%)	99 (2%)	195 (5%)	412 (13%)	717 (5%)
Liver/Pancreas	1 (<1%)	18 (<1%)	68 (2%)	161 (4%)	282 (9%)	530 (4%)
Respiratory	1 (<1%)	35 (1%)	69 (2%)	136 (4%)	293 (9%)	534 (4%)
Endocrine	0 (<1%)	5 (<1%)	45 (1%)	157 (4%)	261 (8%)	468 (3%)

Source: Data from the Dog Aging Project for N=13618 purebred dogs included in the 2020 data release.

Table 2: Proportion with Disease History by Weight Category

	<10kg	10 to <20kg	20 to <30kg	30 to <40kg	>=40kg	Overall
Disease	N=3200	N=2495	N=3418	N=2893	N=1612	N=13618
Skin	842 (26%)	645 (26%)	916 (27%)	870 (30%)	536 (33%)	3809 (28%)
Infection/Parasites	569 (18%)	609 (24%)	949 (28%)	773 (27%)	439 (27%)	3339 (25%)
Bone/Orthopedic	650 (20%)	447 (18%)	585 (17%)	646 (22%)	342 (21%)	2670 (20%)
Gastrointestinal	485 (15%)	371 (15%)	489 (14%)	426 (15%)	265 (16%)	2036 (15%)
Ocular	647 (20%)	402 (16%)	456 (13%)	323 (11%)	181 (11%)	2009 (15%)
Ear/Nose/Throat	495 (15%)	332 (13%)	402 (12%)	442 (15%)	253 (16%)	1924 (14%)
Kidney/Urinary	313 (10%)	213 (9%)	279 (8%)	215 (7%)	100 (6%)	1120 (8%)
Cancer/Tumors	163 (5%)	144 (6%)	243 (7%)	232 (8%)	89 (6%)	871 (6%)
Cardiac	373 (12%)	166 (7%)	151 (4%)	102 (4%)	42 (3%)	834 (6%)
Brain/Neurologic	233 (7%)	123 (5%)	159 (5%)	136 (5%)	66 (4%)	717 (5%)
Liver/Pancreas	253 (8%)	94 (4%)	82 (2%)	71 (2%)	30 (2%)	530 (4%)
Respiratory	218 (7%)	83 (3%)	98 (3%)	84 (3%)	51 (3%)	534 (4%)
Endocrine	116 (4%)	79 (3%)	116 (3%)	106 (4%)	51 (3%)	468 (3%)

Source: Data from the Dog Aging Project for N=13618 purebred dogs included in the 2020 data release.

Table 3: Association of Age and Weight with Lifetime Prevalence (Part 1: Conditions Positively Associated with Weight from Model 1)

		Model 1[a]			Model 2[b]			Model 3[c]		
Condition	Characteristic	PR	95% CI	p-value	PR	95% CI	p-value	PR	95% CI	p-value
Skin	Age	1.32	(1.28, 1.35)	<0.001	1.32	(1.28, 1.35)	<0.001	1.29	(1.26, 1.33)	<0.001
	Weight	1.15	(1.12, 1.18)	<0.001	1.15	(1.12, 1.18)	<0.001	1.15	(1.12, 1.18)	<0.001
	Age * Weight	-	-	-	0.98	(0.96, 1.01)	0.17	0.98	(0.96, 1.01)	0.18
Infection/Parasites	Age	0.98	(0.95, 1.01)	0.27	0.99	(0.96, 1.02)	0.42	0.98	(0.95, 1.01)	0.14
	Weight	1.12	(1.09, 1.15)	<0.001	1.12	(1.09, 1.15)	<0.001	1.12	(1.09, 1.15)	<0.001
	Age * Weight	-	-	-	1.03	(1.00, 1.06)	0.03	1.03	(1.00, 1.06)	0.03
Bone/Orthopedic	Age	2.04	(1.97, 2.11)	<0.001	2.05	(1.98, 2.13)	<0.001	2.00	(1.93, 2.08)	<0.001
	Weight	1.22	(1.18, 1.27)	<0.001	1.18	(1.13, 1.23)	<0.001	1.18	(1.14, 1.23)	<0.001
	Age * Weight	-	-	-	1.07	(1.03, 1.11)	<0.001	1.07	(1.03, 1.11)	<0.01
Gastrointestinal	Age	1.22	(1.18, 1.27)	<0.001	1.22	(1.17, 1.27)	<0.001	1.21	(1.16, 1.26)	<0.001
	Weight	1.05	(1.01, 1.09)	0.03	1.05	(1.01, 1.09)	0.02	1.04	(1.00, 1.08)	0.06
	Age * Weight	-	-	-	0.99	(0.95, 1.03)	0.58	0.99	(0.95, 1.03)	0.64
Ear/Nose/Throat	Age	1.74	(1.67, 1.82)	<0.001	1.72	(1.64, 1.79)	<0.001	1.70	(1.63, 1.78)	<0.001
	Weight	1.16	(1.12, 1.21)	<0.001	1.20	(1.15, 1.26)	<0.001	1.20	(1.15, 1.26)	<0.001
	Age * Weight	-	-	-	0.91	(0.87, 0.94)	<0.001	0.91	(0.87, 0.94)	<0.001
Cancer/Tumors	Age	2.59	(2.43, 2.76)	<0.001	2.58	(2.42, 2.76)	<0.001	2.54	(2.37, 2.71)	<0.001
	Weight	1.37	(1.30, 1.45)	<0.001	1.30	(1.22, 1.39)	<0.001	1.32	(1.23, 1.41)	<0.001
	Age * Weight	-	-	-	1.08	(1.02, 1.14)	0.01	1.07	(1.01, 1.13)	0.02
Brain/Neurologic	Age	2.57	(2.39, 2.77)	<0.001	2.61	(2.42, 2.83)	<0.001	2.61	(2.41, 2.83)	<0.001
	Weight	1.08	(0.99, 1.17)	0.07	1.01	(0.91, 1.12)	0.83	1.00	(0.90, 1.10)	0.96
	Age * Weight	-	-	-	1.08	(1.00, 1.17)	0.04	1.09	(1.01, 1.17)	0.03
Endocrine	Age	2.61	(2.41, 2.83)	<0.001	2.62	(2.41, 2.84)	<0.001	2.60	(2.39, 2.82)	<0.001
	Weight	1.27	(1.16, 1.38)	<0.001	1.24	(1.12, 1.36)	<0.001	1.23	(1.12, 1.35)	<0.001
	Age * Weight	-	-	-	1.03	(0.96, 1.11)	0.39	1.04	(0.96, 1.11)	0.33

Source: Data from the Dog Aging Project for N=13618 purebred dogs included in the 2020 data release.

Note: Age (years) and Weight (kg) are standardized by subtracting their means 7, 24 and dividing by their standard deviations 4, 14.

[a] A model with the main effects of age and weight.

[b] A model with the main effects of age, weight, and the interaction.

[c] A model with the main effects of variables in Model 2 plus adjusted for sex and geographic region.

Table 4: Association of Age and Weight with Lifetime Prevalence (Part 2: Conditions Negatively Associated or Not Associated with Weight from Model 1).

		Model 1[a]			Model 2[b]			Model 3[c]		
Condition	Characteristic	PR	95% CI	p-value	PR	95% CI	p-value	PR	95% CI	p-value
Ocular	Age	1.86	(1.79, 1.94)	<0.001	1.79	(1.71, 1.87)	<0.001	1.78	(1.70, 1.86)	<0.001
	Weight	0.93	(0.89, 0.97)	<0.01	0.98	(0.93, 1.03)	0.46	0.98	(0.93, 1.03)	0.33
	Age * Weight	-	-	-	0.88	(0.85, 0.92)	<0.001	0.88	(0.85, 0.92)	<0.001
Kidney/Urinary	Age	1.73	(1.64, 1.83)	<0.001	1.73	(1.63, 1.83)	<0.001	1.68	(1.59, 1.79)	<0.001
	Weight	1.01	(0.95, 1.07)	0.71	1.02	(0.96, 1.08)	0.59	1.07	(1.00, 1.14)	0.04
	Age * Weight	-	-	-	0.98	(0.93, 1.04)	0.56	0.95	(0.90, 1.01)	0.09
Cardiac	Age	2.12	(2.00, 2.25)	<0.001	2.00	(1.86, 2.16)	<0.001	2.02	(1.87, 2.17)	<0.001
	Weight	0.70	(0.64, 0.76)	<0.001	0.75	(0.68, 0.82)	<0.001	0.73	(0.67, 0.81)	<0.001
	Age * Weight	-	-	-	0.90	(0.84, 0.97)	<0.01	0.91	(0.84, 0.98)	<0.01
Liver/Pancreas	Age	2.00	(1.86, 2.16)	<0.001	1.94	(1.78, 2.12)	<0.001	1.91	(1.74, 2.09)	<0.001
	Weight	0.69	(0.62, 0.77)	<0.001	0.71	(0.63, 0.81)	<0.001	0.72	(0.63, 0.81)	<0.001
	Age * Weight	-	-	-	0.94	(0.86, 1.04)	0.22	0.94	(0.85, 1.03)	0.17
Respiratory	Age	2.06	(1.89, 2.24)	<0.001	2.14	(1.95, 2.35)	<0.001	2.13	(1.94, 2.34)	<0.001
	Weight	0.86	(0.78, 0.96)	<0.01	0.81	(0.71, 0.92)	<0.01	0.80	(0.70, 0.91)	<0.001
	Age * Weight	-	-	-	1.11	(1.00, 1.23)	0.05	1.11	(1.00, 1.23)	0.04

Source: Data from the Dog Aging Project for N=13618 purebred dogs included in the 2020 data release.
Note: Age (years) and Weight (kg) are standardized by subtracting their means 7, 24 and dividing by their standard deviations 4, 14.

[a] A model with the main effects of age and weight.

[b] A model with the main effects of age, weight, and the interaction.

[c] A model with the main effects of variables in Model 2 plus adjusted for sex and geographic region.

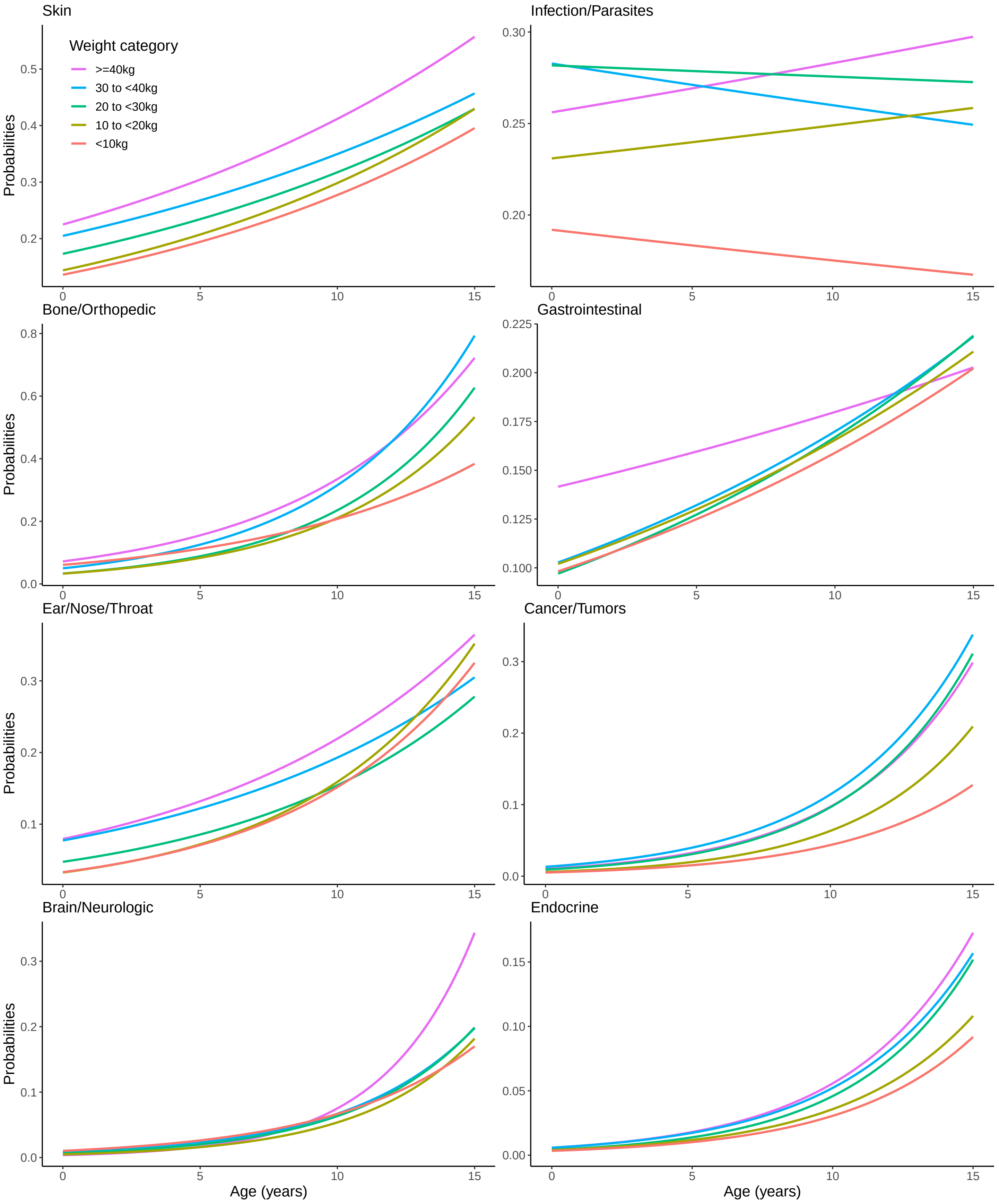

Figure 1: Results from Model 2 with Continuous Age by Weight Category (Part 1: Conditions Positively Associated with Weight from Model 1): Data from the Dog Aging Project for N=13618 purebred dogs included in the 2020 data release.

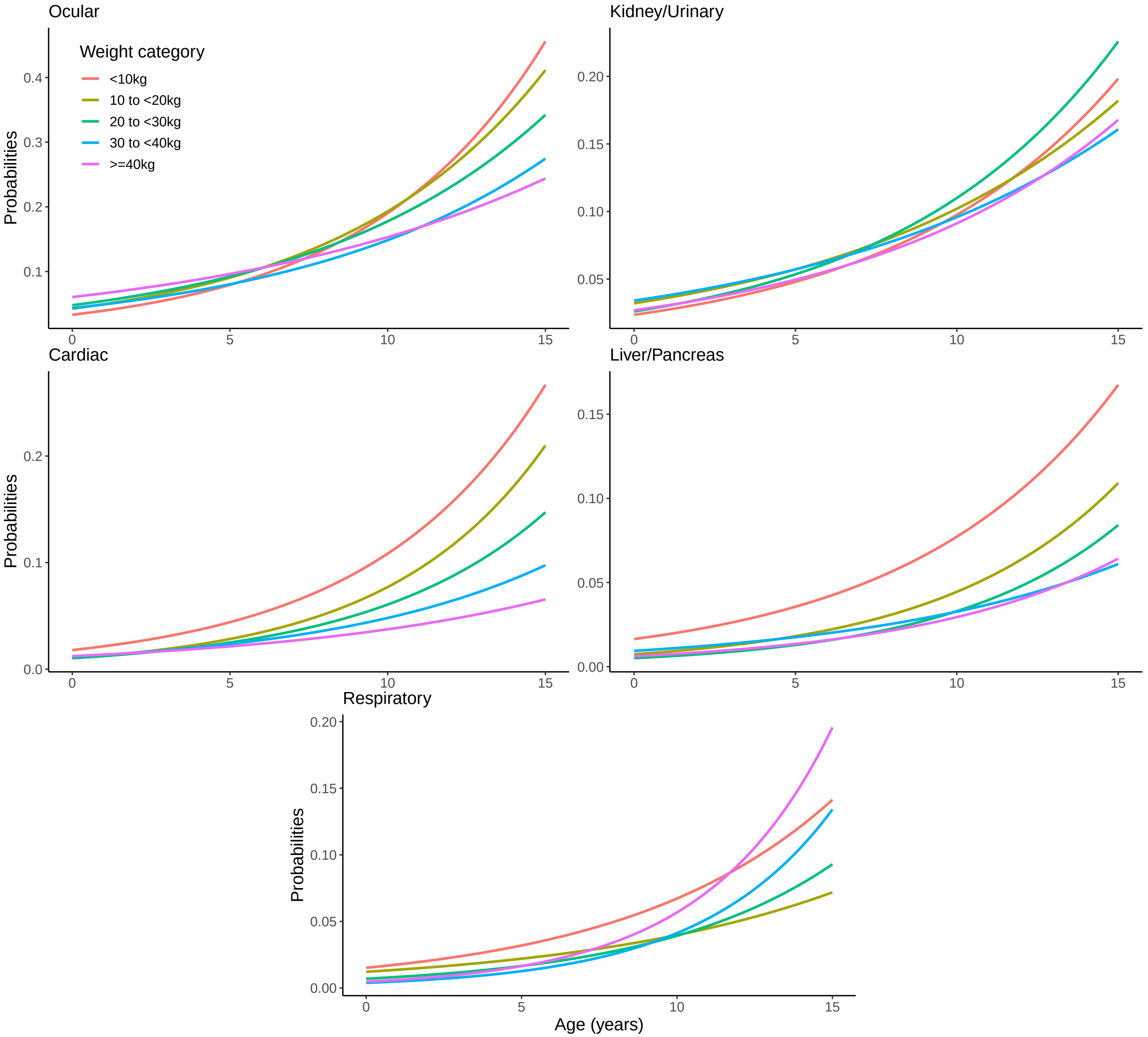

Figure 2: Results from Model 2 with Continuous Age by Weight Category (Part 2: Conditions Negatively Associated or Not Associated with Weight from Model 1): Data from the Dog Aging Project for N=13618 purebred dogs included in the 2020 data release.
