# Supplementary material for: Dog size and patterns of disease history across the canine age spectrum: Results from the Dog Aging Project
Source: PLoS One. 2024 Jan 17;19(1):e0295840. doi: 10.1371/journal.pone.0295840 (PMC10793924; doi:10.1371/journal.pone.0295840)
Supplement: S2 Appendix — (PDF) [file pone.0295840.s003.pdf]

Analysis using subsets of all mixed breed dogs

Table 1: Proportion with Disease History by Age

Disease	Puppy (<1yr) N=252	Adolescent (1 to <3yr) N=2139	Young Adult (3 to <7yr) N=4231	Older Adult (7 to <11yr) N=3980	Senior (>=11yr) N=3321	Overall N=13923
Skin	19 (8%)	380 (18%)	1124 (27%)	1358 (34%)	1225 (37%)	4106 (29%)
Infection/Parasites	71 (28%)	651 (30%)	1257 (30%)	1103 (28%)	918 (28%)	4000 (29%)
Bone/Orthopedic	4 (2%)	107 (5%)	415 (10%)	837 (21%)	1254 (38%)	2617 (19%)
Gastrointestinal	18 (7%)	206 (10%)	501 (12%)	580 (15%)	573 (17%)	1878 (13%)
Ocular	18 (7%)	129 (6%)	237 (6%)	387 (10%)	845 (25%)	1616 (12%)
Ear/Nose/Throat	8 (3%)	126 (6%)	330 (8%)	422 (11%)	759 (23%)	1645 (12%)
Kidney/Urinary	2 (<1%)	72 (3%)	189 (4%)	262 (7%)	476 (14%)	1001 (7%)
Cancer/Tumors	0 (<1%)	8 (<1%)	90 (2%)	280 (7%)	502 (15%)	880 (6%)
Cardiac	3 (1%)	17 (<1%)	71 (2%)	193 (5%)	449 (14%)	733 (5%)
Brain/Neurologic	2 (<1%)	14 (<1%)	89 (2%)	164 (4%)	338 (10%)	607 (4%)
Liver/Pancreas	1 (<1%)	6 (<1%)	57 (1%)	116 (3%)	260 (8%)	440 (3%)
Respiratory	3 (1%)	18 (<1%)	63 (1%)	106 (3%)	226 (7%)	416 (3%)
Endocrine	0 (<1%)	3 (<1%)	32 (<1%)	153 (4%)	257 (8%)	445 (3%)

Source: Data from the Dog Aging Project for N=13923 mixed breed dogs included in the 2020 data release.

Table 2: Proportion with Disease History by Weight Category

Disease	<10kg N=3007	10 to <20kg N=3118	20 to <30kg N=4801	30 to <40kg N=2274	>=40kg N=723	Overall N=13923
Skin	786 (26%)	886 (28%)	1453 (30%)	751 (33%)	230 (32%)	4106 (29%)
Infection/Parasites	617 (21%)	984 (32%)	1492 (31%)	705 (31%)	202 (28%)	4000 (29%)
Bone/Orthopedic	537 (18%)	484 (16%)	876 (18%)	535 (24%)	185 (26%)	2617 (19%)
Gastrointestinal	407 (14%)	415 (13%)	630 (13%)	323 (14%)	103 (14%)	1878 (13%)
Ocular	432 (14%)	398 (13%)	478 (10%)	248 (11%)	60 (8%)	1616 (12%)
Ear/Nose/Throat	332 (11%)	381 (12%)	513 (11%)	310 (14%)	109 (15%)	1645 (12%)
Kidney/Urinary	217 (7%)	239 (8%)	360 (7%)	154 (7%)	31 (4%)	1001 (7%)
Cancer/Tumors	110 (4%)	183 (6%)	322 (7%)	196 (9%)	69 (10%)	880 (6%)
Cardiac	298 (10%)	196 (6%)	150 (3%)	75 (3%)	14 (2%)	733 (5%)
Brain/Neurologic	129 (4%)	155 (5%)	187 (4%)	99 (4%)	37 (5%)	607 (4%)
Liver/Pancreas	140 (5%)	115 (4%)	113 (2%)	59 (3%)	13 (2%)	440 (3%)
Respiratory	167 (6%)	86 (3%)	109 (2%)	40 (2%)	14 (2%)	416 (3%)
Endocrine	78 (3%)	111 (4%)	148 (3%)	70 (3%)	38 (5%)	445 (3%)

Source: Data from the Dog Aging Project for N=13923 mixed breed dogs included in the 2020 data release.

Table 3: Association of Age and Weight with Lifetime Prevalence (Part 1: Conditions Positively Associated with Weight from Model 1)

		Model 1[a]			Model 2[b]			Model 3[c]		
Condition	Characteristic	PR	95% CI	p-value	PR	95% CI	p-value	PR	95% CI	p-value
Skin	Age	1.27	(1.24, 1.30)	<0.001	1.26	(1.23, 1.29)	<0.001	1.25	(1.22, 1.29)	<0.001
	Weight	1.10	(1.07, 1.13)	<0.001	1.11	(1.08, 1.13)	<0.001	1.10	(1.08, 1.13)	<0.001
	Age * Weight	-	-	-	0.97	(0.95, 0.99)	0.01	0.97	(0.95, 0.99)	0.02
Infection/Parasites	Age	0.96	(0.93, 0.98)	<0.01	0.96	(0.93, 0.98)	<0.01	0.95	(0.93, 0.98)	<0.001
	Weight	1.09	(1.07, 1.12)	<0.001	1.10	(1.07, 1.12)	<0.001	1.09	(1.07, 1.12)	<0.001
	Age * Weight	-	-	-	1.04	(1.01, 1.06)	<0.01	1.04	(1.01, 1.07)	<0.01
Bone/Orthopedic	Age	1.95	(1.89, 2.02)	<0.001	1.95	(1.89, 2.02)	<0.001	1.94	(1.88, 2.01)	<0.001
	Weight	1.21	(1.17, 1.25)	<0.001	1.14	(1.09, 1.20)	<0.001	1.14	(1.09, 1.20)	<0.001
	Age * Weight	-	-	-	1.10	(1.06, 1.14)	<0.001	1.10	(1.06, 1.15)	<0.001
Gastrointestinal	Age	1.23	(1.18, 1.28)	<0.001	1.22	(1.17, 1.27)	<0.001	1.21	(1.17, 1.27)	<0.001
	Weight	1.01	(0.97, 1.06)	0.54	1.02	(0.97, 1.06)	0.47	1.01	(0.97, 1.05)	0.72
	Age * Weight	-	-	-	0.99	(0.95, 1.03)	0.54	0.99	(0.95, 1.03)	0.61
Ear/Nose/Throat	Age	1.78	(1.70, 1.87)	<0.001	1.77	(1.68, 1.85)	<0.001	1.76	(1.68, 1.85)	<0.001
	Weight	1.13	(1.08, 1.18)	<0.001	1.20	(1.14, 1.26)	<0.001	1.20	(1.14, 1.26)	<0.001
	Age * Weight	-	-	-	0.88	(0.85, 0.92)	<0.001	0.88	(0.85, 0.92)	<0.001
Cancer/Tumors	Age	2.51	(2.37, 2.67)	<0.001	2.50	(2.35, 2.65)	<0.001	2.50	(2.36, 2.66)	<0.001
	Weight	1.44	(1.36, 1.52)	<0.001	1.38	(1.29, 1.48)	<0.001	1.39	(1.30, 1.49)	<0.001
	Age * Weight	-	-	-	1.05	(1.00, 1.11)	0.07	1.05	(1.00, 1.12)	0.07
Brain/Neurologic	Age	2.34	(2.16, 2.53)	<0.001	2.35	(2.17, 2.55)	<0.001	2.37	(2.19, 2.57)	<0.001
	Weight	1.10	(1.02, 1.19)	0.02	1.07	(0.95, 1.19)	0.26	1.05	(0.94, 1.17)	0.39
	Age * Weight	-	-	-	1.04	(0.95, 1.13)	0.37	1.05	(0.97, 1.14)	0.25
Endocrine	Age	2.58	(2.39, 2.79)	<0.001	2.58	(2.38, 2.79)	<0.001	2.59	(2.39, 2.80)	<0.001
	Weight	1.27	(1.16, 1.38)	<0.001	1.28	(1.16, 1.43)	<0.001	1.27	(1.15, 1.41)	<0.001
	Age * Weight	-	-	-	0.98	(0.91, 1.06)	0.63	0.99	(0.92, 1.06)	0.75

Source: Data from the Dog Aging Project for N=13923 mixed breed dogs included in the 2020 data release.
Note: Age (years) and Weight (kg) are standardized by subtracting their means 7, 21 and dividing by their standard deviations 4, 11.

[a] A model with the main effects of age and weight.
[b] A model with the main effects of age, weight, and the interaction.
[c] A model with the main effects of variables in Model 2 plus adjusted for sex and geographic region.

Table 4: Association of Age and Weight with Lifetime Prevalence (Part 2: Conditions Negatively Associated or Not Associated with Weight from Model 1).

		Model 1[a]			Model 2[b]			Model 3[c]		
Condition	Characteristic	PR	95% CI	p-value	PR	95% CI	p-value	PR	95% CI	p-value
Ocular	Age	1.86	(1.78, 1.95)	<0.001	1.83	(1.75, 1.93)	<0.001	1.84	(1.75, 1.93)	<0.001
	Weight	0.91	(0.87, 0.96)	<0.001	0.95	(0.89, 1.00)	0.05	0.94	(0.89, 1.00)	0.04
	Age * Weight	-	-	-	0.94	(0.90, 0.99)	0.01	0.94	(0.90, 0.99)	0.01
Kidney/Urinary	Age	1.80	(1.70, 1.90)	<0.001	1.78	(1.68, 1.89)	<0.001	1.76	(1.65, 1.86)	<0.001
	Weight	0.99	(0.93, 1.04)	0.62	1.01	(0.95, 1.07)	0.74	1.07	(1.00, 1.15)	0.04
	Age * Weight	-	-	-	0.96	(0.91, 1.01)	0.10	0.93	(0.88, 0.98)	0.01
Cardiac	Age	2.29	(2.15, 2.44)	<0.001	2.27	(2.09, 2.46)	<0.001	2.28	(2.10, 2.47)	<0.001
	Weight	0.63	(0.58, 0.69)	<0.001	0.64	(0.58, 0.71)	<0.001	0.64	(0.57, 0.71)	<0.001
	Age * Weight	-	-	-	0.98	(0.91, 1.07)	0.68	0.99	(0.91, 1.08)	0.86
Liver/Pancreas	Age	2.25	(2.07, 2.44)	<0.001	2.34	(2.14, 2.56)	<0.001	2.34	(2.14, 2.57)	<0.001
	Weight	0.82	(0.74, 0.91)	<0.001	0.75	(0.66, 0.86)	<0.001	0.76	(0.66, 0.86)	<0.001
	Age * Weight	-	-	-	1.11	(1.01, 1.23)	0.03	1.12	(1.02, 1.24)	0.02
Respiratory	Age	1.98	(1.81, 2.17)	<0.001	1.97	(1.75, 2.21)	<0.001	1.97	(1.76, 2.22)	<0.001
	Weight	0.71	(0.63, 0.80)	<0.001	0.71	(0.62, 0.82)	<0.001	0.71	(0.62, 0.82)	<0.001
	Age * Weight	-	-	-	0.99	(0.88, 1.12)	0.88	1.00	(0.88, 1.13)	0.98

Source: Data from the Dog Aging Project for N=13923 mixed breed dogs included in the 2020 data release.

Note: Age (years) and Weight (kg) are standardized by subtracting their means 7, 21 and dividing by their standard deviations 4, 11.

[a] A model with the main effects of age and weight.

[b] A model with the main effects of age, weight, and the interaction.

[c] A model with the main effects of variables in Model 2 plus adjusted for sex and geographic region.

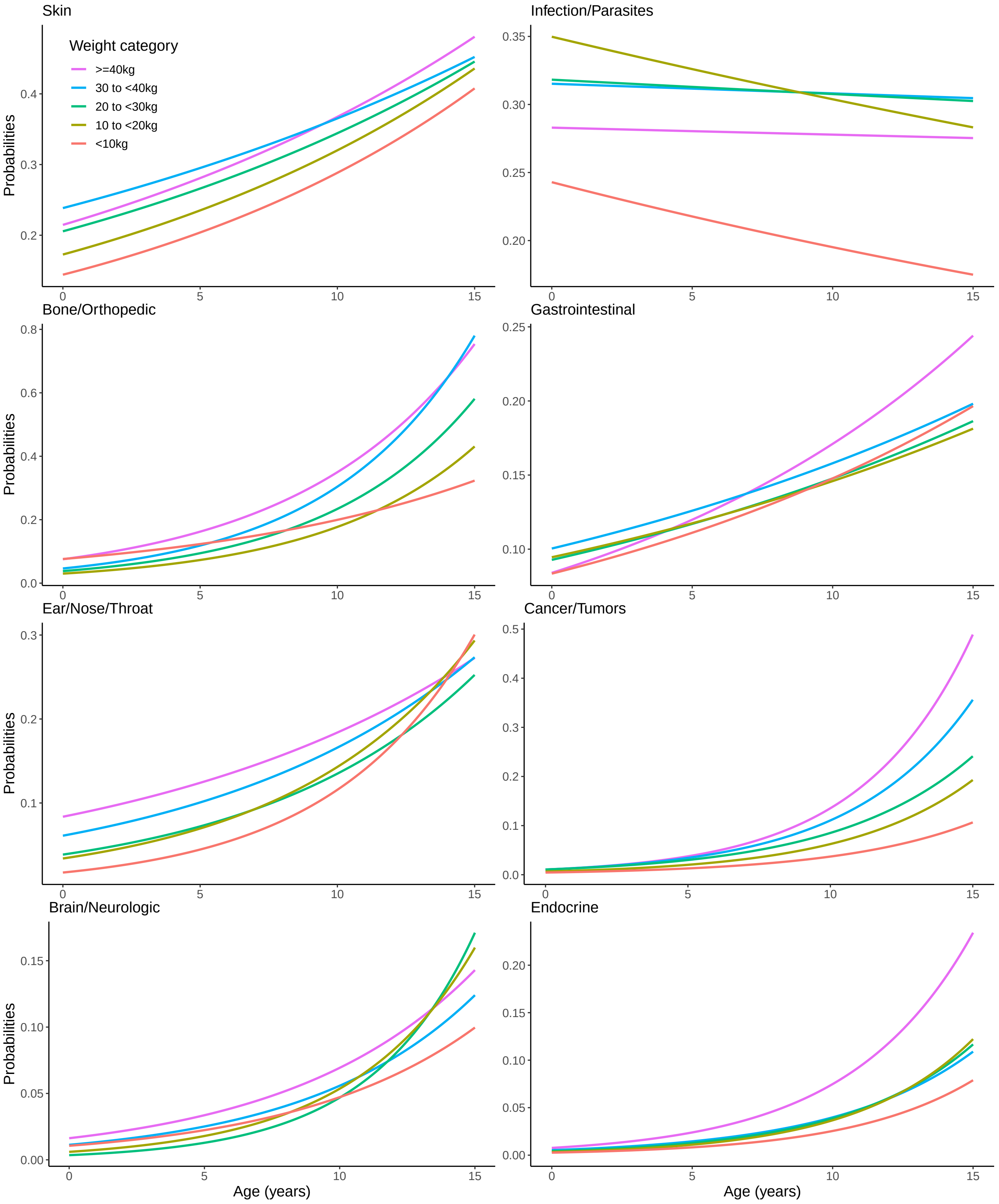

Figure 1: Results from Model 2 with Continuous Age by Weight Category (Part 1: Conditions Positively Associated with Weight from Model 1): Data from the Dog Aging Project for N=13923 mixed breed dogs included in the 2020 data release.

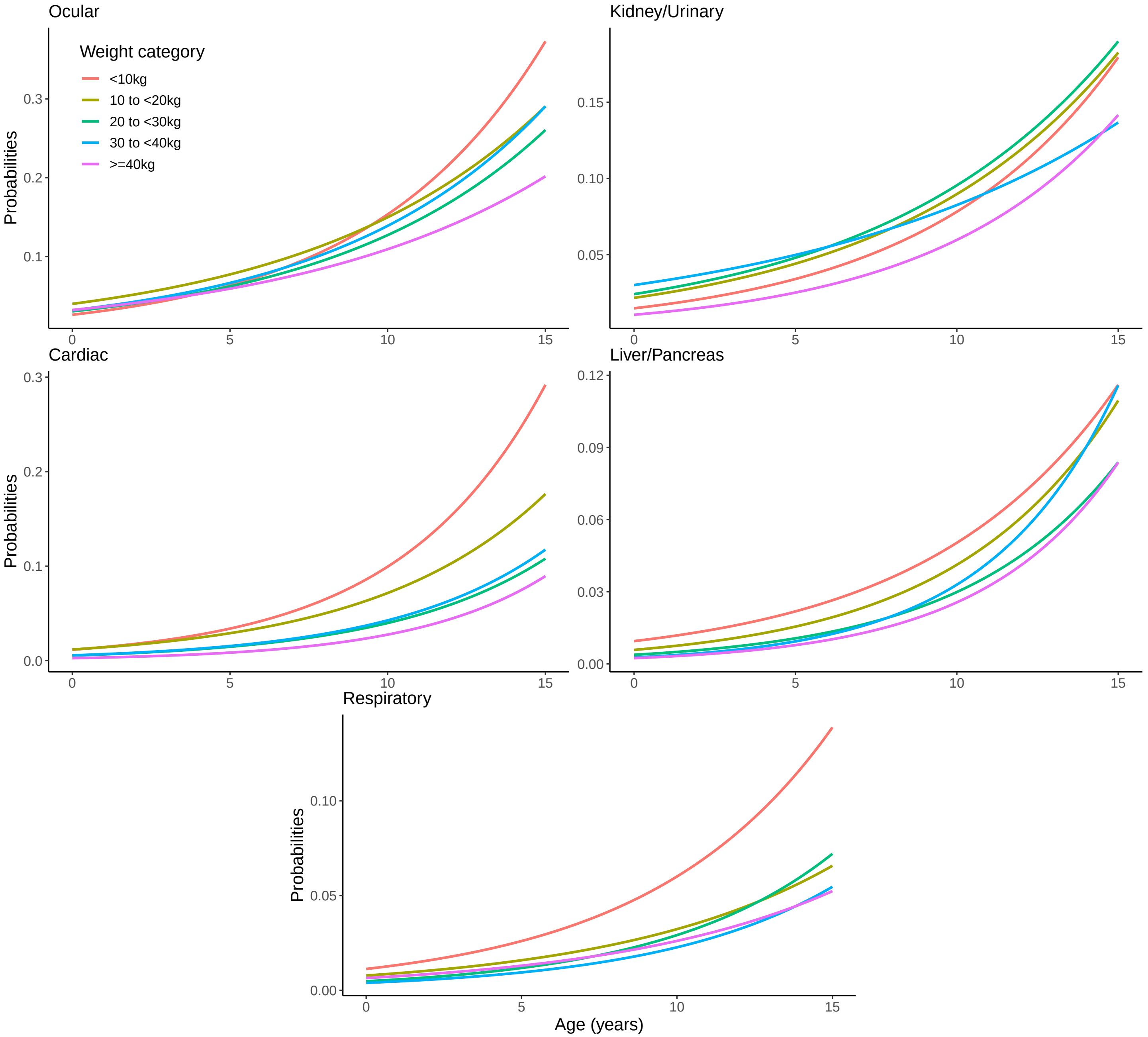

Figure 2: Results from Model 2 with Continuous Age by Weight Category (Part 2: Conditions Negatively Associated or Not Associated with Weight from Model 1): Data from the Dog Aging Project for N=13923 mixed breed dogs included in the 2020 data release.
